# Comparative Analysis of Online Health Queries Originating From Personal Computers and Smart Devices on a Consumer Health Information Portal

**DOI:** 10.2196/jmir.3186

**Published:** 2014-07-04

**Authors:** Ashutosh Jadhav, Donna Andrews, Alexander Fiksdal, Ashok Kumbamu, Jennifer B McCormick, Andrew Misitano, Laurie Nelsen, Euijung Ryu, Amit Sheth, Stephen Wu, Jyotishman Pathak

**Affiliations:** ^1^Knoesis CeneterComputer Science and EngineeringWright State UniversityDayton, OHUnited States; ^2^Mayo ClinicRochester, MNUnited States; ^3^Division of Biomedical Statistics and InformaticsDepartment of Health Sciences ResearchMayo ClinicRochester, MNUnited States

**Keywords:** online health information seeking, health information search, eHealth, mHealth, search query analysis, health search log, mobile health, health seeking behavior

## Abstract

**Background:**

The number of people using the Internet and mobile/smart devices for health information seeking is increasing rapidly. Although the user experience for online health information seeking varies with the device used, for example, smart devices (SDs) like smartphones/tablets versus personal computers (PCs) like desktops/laptops, very few studies have investigated how online health information seeking behavior (OHISB) may differ by device.

**Objective:**

The objective of this study is to examine differences in OHISB between PCs and SDs through a comparative analysis of large-scale health search queries submitted through Web search engines from both types of devices.

**Methods:**

Using the Web analytics tool, IBM NetInsight OnDemand, and based on the type of devices used (PCs or SDs), we obtained the most frequent health search queries between June 2011 and May 2013 that were submitted on Web search engines and directed users to the Mayo Clinic’s consumer health information website. We performed analyses on “Queries with considering repetition counts (QwR)” and “Queries without considering repetition counts (QwoR)”. The dataset contains (1) 2.74 million and 3.94 million QwoR, respectively for PCs and SDs, and (2) more than 100 million QwR for both PCs and SDs. We analyzed structural properties of the queries (length of the search queries, usage of query operators and special characters in health queries), types of search queries (keyword-based, wh-questions, yes/no questions), categorization of the queries based on health categories and information mentioned in the queries (gender, age-groups, temporal references), misspellings in the health queries, and the linguistic structure of the health queries.

**Results:**

Query strings used for health information searching via PCs and SDs differ by almost 50%. The most searched health categories are “Symptoms” (1 in 3 search queries), “Causes”, and “Treatments & Drugs”. The distribution of search queries for different health categories differs with the device used for the search. Health queries tend to be longer and more specific than general search queries. Health queries from SDs are longer and have slightly fewer spelling mistakes than those from PCs. Users specify words related to women and children more often than that of men and any other age group. Most of the health queries are formulated using keywords; the second-most common are wh- and yes/no questions. Users ask more health questions using SDs than PCs. Almost all health queries have at least one noun and health queries from SDs are more descriptive than those from PCs.

**Conclusions:**

This study is a large-scale comparative analysis of health search queries to understand the effects of device type (PCs vs SDs) used on OHISB. The study indicates that the device used for online health information search plays an important role in shaping how health information searches by consumers and patients are executed.

## Introduction

### Background

Limited health literacy is associated with higher rates of hospitalizations, poorer health, and higher mortality [1,2]. Online health information plays a vital role in improving health literacy and helps online health information seekers (OHIS) make more informed health decisions. Over the past decade, Internet literacy and the number of Internet users have increased significantly [3-5]. With the growing availability of eHealth resources [6,7], consumers are increasingly using the Internet to seek health-related information. According to a 2013 Pew Survey [4], one in three American adults has gone online to find information about a specific medical condition. With the recent exponential increase in usage of smart devices (SDs), like smartphones or tablets, the percentage of people using smart devices to search for health information is also growing rapidly [8,9].

While there is some evidence [10] that the experience of online information searching varies depending on the device used (eg, smart devices vs personal computers or laptops [PCs]), little is known about how device choice impacts the structure of health information search queries generated by users. Understanding the effects of the device used (SDs vs PCs) for health information search would help us to acquire more insights into online health information seeking behavior (OHISB). Such knowledge can be applied to improve the search experience and to develop more advanced next-generation knowledge and content delivery systems.

One of the most common ways to seek online health Information is via Web search engines such as Google, Yahoo!, etc [4]. According to the Pew Survey [4], approximately 8 in 10 online health inquiries initiate from a search engine. A typical online health information search process starts with the formulation of a health search query based on an OHIS information need. This query is typically submitted to a Web search engine, which subsequently leads to visiting one or more websites recommended by the search engine. In this paper, we study the effect of the devices used for health information search, concentrating on what information users search for and how health search queries are formulated.

Using the Mayo Clinic website’s Web analytics tool (IBM NetInsight OnDemand [11]) and based on the type of devices used (PCs or SDs), we obtained the most frequent health search queries submitted from Web search engines that direct traffic to the Mayo Clinic webpages [12]. We selected search queries that are in the English language and collected between June 2011 and May 2013. We analyzed structural properties, types (keywords, wh-question, yes/no-questions), misspellings, and the linguistic structure of the health queries. We further categorized them based on health categories and demographic information mentioned (gender, age group, etc) in the queries. Our analysis suggests that the device used for online health information searching plays a significant role, altering the OHISB.

### Significance of Current Study

Many previous studies have investigated OHISB. Researchers have used several approaches to understand OHISB including (1) focus groups and user surveys [13-20] and (2) analyzing health-related Web search query logs [21-32]. In the studies that involved focus groups and user surveys, researchers have analyzed characteristics associated with OHISB such as how people use the Internet for health information searching, their demographic information (age, gender, education level, etc), devices/Web search engines used for searching, OHISB in specific health conditions, and age groups [13-20]. Although these studies provide important insights into OHISB, their main limitation was the inclusion of a small number of participants (ranging from 100-2000 people). A second approach to studying OHISB is analyzing Web search logs from the health domain. Several previous studies have analyzed health search query logs with diverse objectives, such as health/epidemic surveillance [33-39], PubMed usage [40,41], and OHISB [21-32]. The studies focusing on OHISB [21-32] have studied a variety of aspects of health query logs, such as query length, health categories, relationship between OHISB and health care utilization [24], changes in health behavior with type of disease [21], and changes in OHISB with disease escalation from symptoms to serious illness [22,23].

Although the user experience for online health information searching varies with the device used (PCs/SDs) [10], there is a dearth of work relating OHISB with the device used for searching. In this study, we address this problem by analyzing large-scale health queries for both PCs and SDs to understand the effects of device type (PCs vs SDs) used for online health information seeking. Previous studies in generic search query log analysis have determined the importance of understanding linguistic structure of search queries as it has implications on information retrieval using Web search engines [42,43]. One of the contributions of this study is a comparative analysis of linguistic structure of health search queries from PCs and SDs. This study provides useful and interesting findings that can be leveraged in multiple ways. Some of the potential beneficiaries are (1) Web search engines: to understand health search query structure and complexity, and the occurrence of popular health categories for PCs and SDs to improve query performance and accuracy for health information retrieval systems, (2) Websites that provide health information: to better understand online health information seekers’ health information need, and better organize health information content for PCs and SDs users, (3) Health care providers: to better understand their patients and their health information interests, (4) Health care-centric application developers: to better understand OHISB for PCs and SDs and build applications around consumer’s health information needs and priorities, and (5) online health information seekers: we anticipate that this work will help empower online health information seekers in their quest for health information and facilitate their health information search efforts by enabling the development of smarter and more sophisticated consumer health information delivery mechanisms.

## Methods

### Data Source

In this study, we collected health search queries originating from Web search engines (such as Google and Bing) that direct OHISs to the Mayo Clinic’s consumer health information website [12], which is one of the top online health information website within the United States. The Mayo Clinic provides up-to-date, high-quality online health information produced by professional writers and editors. Our recent Web analytics statistics indicate that the Mayo Clinic website is visited by millions of unique visitors on average every day, and around 90% of the incoming traffic originated from Web search engines. The Mayo Clinic website is identical in terms of appearance and functionality for both PCs and SDs using standard Web search engines and Web browsers. This consistency as well as significant traffic to the website provide us with an excellent platform to conduct our study.

### Dataset Creation

The Mayo Clinic website’s Web analytics tool, IBM NetInsight OnDemand [11], keeps detailed information about incoming Web traffic from Web search engines to the Mayo Clinic website. The tool maintains information such as input search query (the original query from a Web search engine that brings an OHIS to the Mayo Clinic website), number of query repetitions (how many times the query has been searched within specified time period), and the visitor’s Operating System (OS). PCs generally use Windows (98, 2000, Xp, Vista, 7, 8), Mac OS X, or Linux (such as Ubuntu and Redhat) operating systems while SDs use iOS (iPhone’s OS), Android, Windows Mobile, and RIM BlackBerry operating systems. Since the Web analytics tool tracks information related to each user’s OS type and individual searches, we are able to differentiate search queries by device type (PCs/SDs).

Using the Web analytics tool, we obtained one data report for each of the most frequent one million (based on the number of query repetitions) anonymized distinct queries in the English language launched from PCs and SDs for each month between June 2011 and May 2013 (24 months), totalling 48 data reports. Each search query appears uniquely in each data report and has an associated number of query repetitions. For each device type (PCs and SDs), we aggregated 24 reports to create a single report with distinct queries. The dataset for PCs has 2.74 million queries, and the dataset for SDs has 3.94 million queries. While aggregating the search queries for PCs and SDs, we combined the repetition counts for each repeated query; for example, if a “diabetes” query has 5 repetitions in 1 month and 10 repetitions in another month, then the total number of repetition for the “diabetes” query is 15. Note that selecting the top queries for 2 years would be an easier approach for dataset creation, but in our case the data reports were available by month, thus we have to aggregate the data for each month to create the final analysis dataset.

### Data Analysis

#### Overview

In this study, we performed analyses on “queries *with considering* repetition counts (QwR)” and “queries *without considering* repetition counts (QwoR)”. Because the analysis performed with only QwR may overrepresent certain queries due to their large number of repetitions, we performed the analysis for both QwoR and QwR. The QwoR count is the same as the number of queries in the dataset. Hence for PCs, we have 2.74 million QwoR, and for SDs we have 3.94 million QwoR. We obtained the QwR count by aggregating number of repetitions for all the queries in the dataset. For both PCs and SDs, we got more than 100 million QwR. Due to Mayo Clinic’s confidentiality policy, we are not able to disclose the exact number of QwR. We are reporting percentages of PC and SD queries.

#### Top Health Queries

The top search queries are the most commonly searched queries. To analyze the top health queries launched from PCs and SDs, we selected the top 100 search queries, from PCs and SDs, based on the descending order of number of query repetitions in the analysis dataset.

#### Health Categories

**Figure 1:**
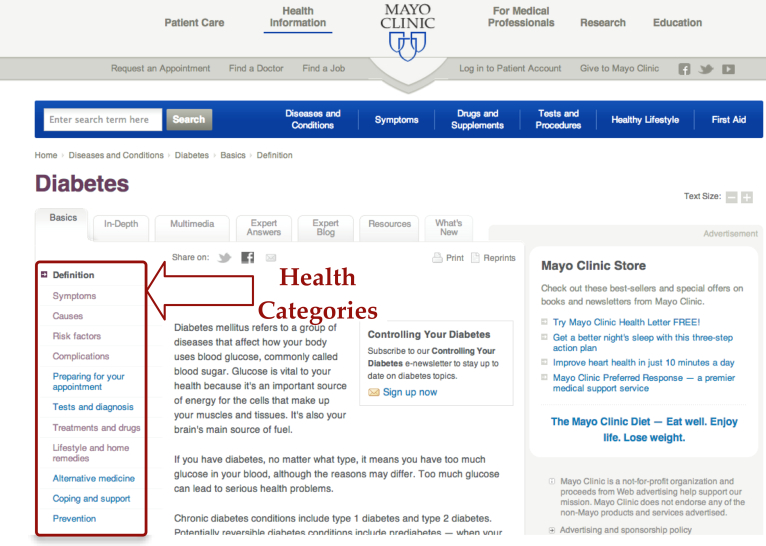
Screenshot of Mayo Clinic website for Diabetes (left-side box highlights organization of health information based on health categories).

To analyze popular health categories that OHISs search for from PCs and SDs, we selected the following 8 health categories corresponding to the organization of health topics on popular health websites (Mayo Clinic, MedlinePlus [44], WebMD [45]): Symptoms, Causes, Complications, Tests and Diagnosis, Treatments and Drugs, Risk Factors, Prevention, Coping and Support. For example, Figure 1 shows different health categories for diabetes on the Mayo Clinic website, where each health category has a separate webpage with detailed information (browsable via navigating the left panel). Based on the semantics of an OHIS’s input search query and a Web search engine’s recommendations, users may land on one of the health category pages on the Mayo Clinic website. For this study, we aggregated all the incoming health search queries between June 2011 and May 2013 that land on a particular health category webpages. For example, we aggregated all the search queries that land on the “Symptoms” webpage for all the diseases and health conditions on the Mayo Clinic website. We analyzed the type of device (PC or SD) used for searches and the number of search queries to each health category.

#### Categorization Based on the Information Mentioned in the Health Queries

In order to understand how often an OHIS mentions gender, age groups, and temporal references in the search queries, we categorized health queries using a dictionary-based approach. For each group, we created a lexicon by going through online English dictionaries [46-48] and a manual evaluation of words. For example, in the “Gender” group we considered Men (Man, men, male, boy, gent, gentleman, gentlemen) and Women (Woman, women, female, girl, ladies, lady). We also considered keywords’ lexical variants; for example, boy, boys, etc. We categorized search queries from PCs and SDs by utilizing the lexicon for each category.

#### Health Query Length

To study the difference in health search query length for queries from PCs and SDs, we calculated search query length by computing the number of words (separated by white space) and the number of characters (excluding white space) in the health queries.

#### Usage of Query Operators and Special Characters

In search queries, query operators (“and”, “or”, “not”, etc) are used to formulate complex queries. In this study, we considered the following operators: AND, OR, +, &, other (NOT, AND NOT, OR NOT, & NOT). Special characters are characters apart from letters (a-z) and digits (0-9). The significance of special characters in a health search query depends on the usage of special characters in the medical domain. For example, OHIS may mention values in different formats, eg, 2.3 ml, 40%, 17-19, or $200 (for the cost of a drug or procedure). We analyzed the usage of search query operators and special characters in health queries based on their usage frequency in the PCs and SDs search queries.

#### Misspellings in Health Queries

OHISs occasionally make spelling mistakes while searching for health information. To analyze the frequency of such errors, we used a dictionary-based approach. We first generated a dictionary of words using the Zyzzyva wordlist [49], the Hunspell dictionary [50], and its medical version (OpenMedSpell [51]), comprising a total of 275,270 unique words. We used this dictionary to check misspellings in health search queries from PCs and SDs.

#### Type of Search Queries

OHISs express their health information need by formulating health search queries on Web search engines. In general, each health search query indicates some health information need. OHISs can express their information need either by formulating search queries using keywords or asking questions (wh-questions and yes/no questions). For this analysis, we considered the following wh-questions (lexicon): “What”, “How”, “?”, “When”, “Why”, and others (“Who” “Where”, “Which”). Note that although “?” does not come under the wh-questions category, we have included it for simplicity. Yes/No questions are usually used to check factual information; for example, whether coffee is bad for the heart. In this analysis, we considered yes/no questions that start with “Can”, “Is”, “Does”, “Do”, “Are”, and others (“Could”, “Should”, “Will”, “Would”). Using the lexicon for wh-questions and yes/no questions, we performed text analysis on the search queries from PCs and SDs to count the number of queries with wh-questions and yes/no questions. Search queries that do not contain any question (wh- or yes/no) are classified as keyword-based. Additionally, for different wh- and yes/no questions, we computed their usage frequency in search queries from PCs and SDs.

#### Linguistic Analysis of Health Queries

Previous studies in generic search query log analysis have identified that understanding the linguistic structure, including phrase identification, entitity spotting and discriptiveness (level of context), of search queries can improve Web Information Retrieval systems [42,43]. However, these efforts have not been applied extensively to health search queries, and hence in order to understand the linguistic structure of health queries, we performed part-of-speech analysis on search queries using Stanford’s POS tagger [52]. For this analysis, we considered nouns, verbs, adjectives and adverbs. We mapped all subtypes in part-of-speech (eg, proper nouns, common nouns, compound nouns) to the main part-of-speech (eg, nouns). We analyzed the usage of different part-of-speech types in health queries based on their usage frequency in the PCs and SDs search queries.

## Results

### Top Health Queries

Most of the top search queries from both PCs and SDs are for symptom descriptions (eg, “lupus symptoms”). Another common way an OHIS searches for health information is by disease name (eg, “Lupus”). Chronic diseases (cancer, cardiovascular disease, diabetes) and diet (Mediterranean diet, gluten free food) are also searched often. Based on the top 100 search queries from PCs and SDs, we found that 48.49% of the search queries are different between PCs and SDs. However, due to the Mayo Clinic business confidentiality, we are not in a position to disclose the actual top search queries and numbers publicly.

### Health Categories

**Figure 2:**
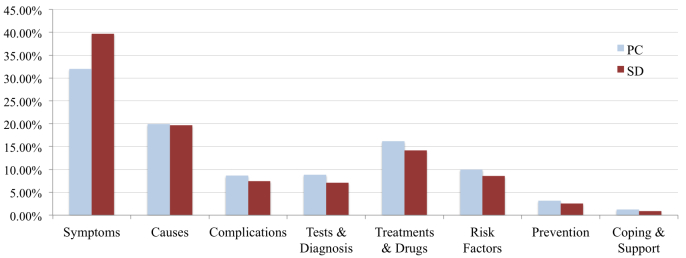
Distribution of the search queries by health categories.

While searching for health information, one in every three OHIS searches for “Symptoms” (Figure 2). Other popular health categories are “Causes” and “Treatments & Drugs”. Our analysis shows that the distribution of search queries for different health categories differs with the device used for the health search. At the same time, both PCs and SDs follow a similar pattern for distribution of the search queries between health categories. The percentage of OHIS searching for “Symptoms” is higher from SDs as compared to that from PCs. While for other health categories, the percentage of queries from PCs is slightly higher than that of SDs. Interestingly, one of the least searched health categories is “Prevention”.

### Categorization Based on the Information Mentioned in the Health Queries

The following are some of our observations based on the information referenced in the search queries (Table 1). The data indicate that the number of search queries mentioning words related to women’s health is considerably higher compared to that of men. This implies that OHIS search for health information specifying women more often. The percentage of OHIS who use words related to “woman” in search queries is higher for PCs compared to SDs. Considering age group–related search queries, more than 60% of the queries are related to children. The percentage of OHIS that mention terms related to children in search queries is much higher for SDs compared to PCs. When considering a mention of the time of day in search queries, terms related to “Night” are mentioned most often (>60%) followed by words related to “Morning”. Very few search queries have words related to “Afternoon” and “Evening”. The percentage of OHIS using words related to “Morning” in search queries is higher for SDs compared to PCs, while the percentage of OHIS mentioning words related to “Night” in search queries is higher for PCs.

**Table 1:** Categorization of health search queries based on the information mentioned in the queries such as gender, age group, and temporal information (June 2011-May 2013).

		Personal computers		Smart device	
		QwoR %	QWR %	QwoR %	QWR %
**Gender**					
	Men	25.62	17.25	28.48	18.17
	Women	74.38	82.75	71.52	81.83
**Age group**					
	Children	66.55	59.60	79.33	74.39
	Teen	7.25	5.08	3.69	2.37
	Adults	18.60	31.68	13.64	21.72
	Elders	7.60	3.64	3.34	1.52
**Temporal**					
	Morning	26.85	29.93	31.93	39.14
	Afternoon/Evening	5.84	4.39	4.10	1.73
	Night	67.31	65.68	63.98	59.13

### Health Query Length

**Figure 3:**
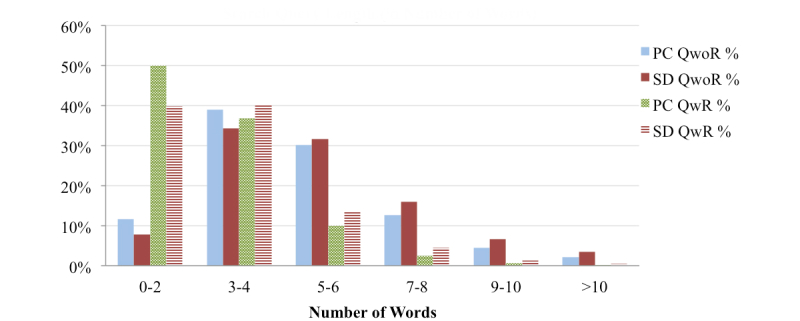
Distribution of the search queries by number of words and number.

**Figure 4:**
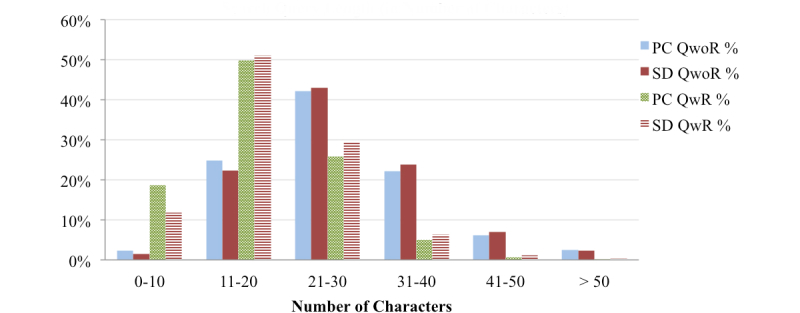
Distribution of the search queries by number of characters.

The average search query length (Figures 3 and Figure 4) for QwoR (PCs: 4.82 words and 26.73 characters; SDs: 5.33 words and 27.41 characters) is much larger than the average length of QwR (PCs: 2.90 words and 17.61 characters; SDs: 3.29 words and 18.86 characters). This indicates that longer search queries result in fewer repetitions, while shorter queries tend to be repeated more often. The analysis, although derived from a limited dataset, implies that in general health search queries tend to be longer than general search queries (not specific to one domain), as the average length of general search query from PCs is 2-2.35 words [53-55] and from SDs is 2.3 words [56]. This potentially indicates that OHISs describe their health information needs in more detail by adding relevant health context to the search query. Surprisingly, the average length of search query from SDs for both QwoR and QwR is slightly larger than queries from PCs.

### Usage of Query Operators and Special Characters

In considering both PCs and SDs, approximately 10% of QwoR and 3% of QwR use at least one query operator. For QwR, the percentage of OHIS who use query operators in search queries is higher for SDs than PCs, while in the case of QWOR it is higher for PCs. AND is the most popular operator, followed by OR and “+”. Overall variations of “and” (AND, &, +) operators comprise more than 90% of operator usage. Considering QwoR, OHIS use AND OR query operators more often from SDs than that from PCs. Considering both PCs and SDs, around 10% of QwoR and 4% of QwR have at least one special character (Table 2). The percentage of OHIS using special characters in search queries is higher for PCs compared to SDs.

**Table 2:** Usage of query operators and special characters (June 11-May 13).

		Personal computers		Smart device	
		QwoR %	QwR %	QwoR %	QwR %
**Number of operators**					
	0	90.08	97.35	90.23	96.53
	>0	9.92	2.65	9.77	3.47
**Query operators usage**					
	AND	78.96	86.53	82.01	85.05
	+	11.24	4.37	6.29	3.08
	OR	6.95	5.20	8.74	6.78
	&	2.63	1.42	2.57	1.28
	Other	0.24	2.49	0.40	3.82
**Special characters**					
	0	89.02	95.66	90.54	96.72
	>0	10.98	4.34	9.46	3.29
**Spelling mistake**					
	0	68.21	87.47	69.07	87.88
	>0	31.80	12.54	30.94	12.12

### Misspellings in Health Queries

For QwoR and QwR, approximately 31% and 12% of queries, respectively, have at least one spelling mistake (Table 2). OHISs make slightly more spelling mistakes while searching health information from PCs than SDs.

### Types of Health Queries

**Figure 5:**
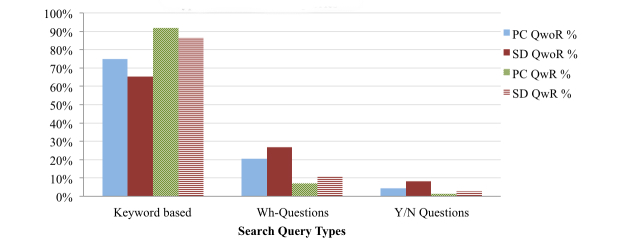
Types of health search queries (how health information need is expressed).

**Figure 6:**
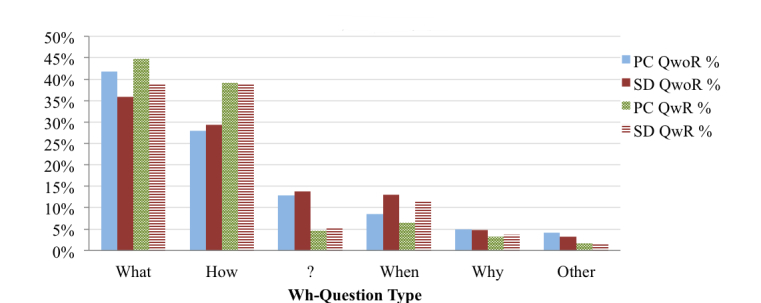
Distribution of the search queries based on type of wh-questions.

**Figure 7:**
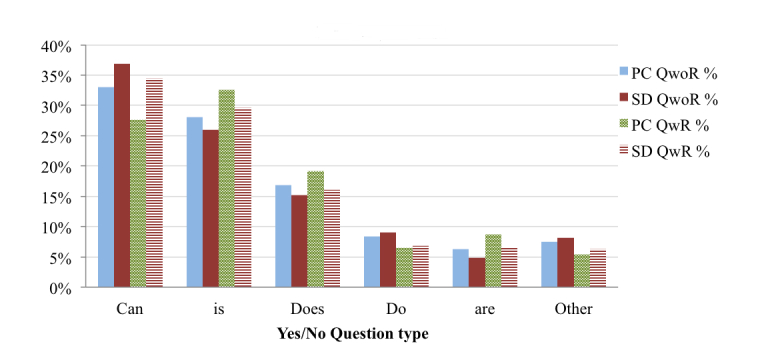
Distribution of the search queries based on type of yes/no questions.

As indicated by the analysis in Figure 5, OHISs predominantly formulate search queries using keywords, though wh-questions and yes/no questions are also substantial. Considering QwoR, OHISs ask more (wh- and yes/no) questions from SDs than PCs. In wh-questions (Figure 6), OHISs mostly use “What” and “How” in the search queries, and both of them generally signify that more descriptive information is needed. OHISs ask more temporal questions (“When”) using SDs than PCs, while OHISs ask more “What” questions using PCs than SDs. In yes/no questions (Figure 7), OHISs generally start search queries with “Can,” “Is”, and “Does”. OHISs ask more yes/no questions starting with “Can” using SDs than using PCs, while the percentage of questions starting with “Is” and “Does” comes more from PCs.

### Linguistic Analysis of Health Queries

In health search queries, nouns typically denote entities like disease names, health categories, etc. Almost all health search queries have at least one noun. In the case of QwR, most of the search queries (>70%) have 1-2 nouns, while in the case of QwoR, most of the search queries (>60%) have 2-3 nouns. There is no considerable difference in noun usage between PCs and SDs. A verb conveys an action or an occurrence, for example “how to control (*verb*) diabetes (*noun*)?”. Considering QwoR, OHIS use at least one verb in 37% of queries from PCs and 47% in queries from SDs. Adverbs are words that modify a verb, an adjective, and another adverb, while an adjective is a “describing” word, giving more information about the object signified; for example, “extremely (*adverb*) bad (*adjective*) stomach (*noun*) pain (*noun*)”. Very few search queries have at least one adverb. Considering QwoR, 45.66% of the queries from PCs and 48.50% of the queries from SDs have at least one adjective. This indicates that the percentage of search queries with at least one verb/adverb/adjective is higher for SDs than for PCs (see Table 3).

**Table 3:** Linguistic analysis of health search queries (June 2011-May 2013).

		Personal computers		Smart device	
		QwoR %	QwR %	QwoR %	QwR %
**Nouns**					
	0	0.96	3.19	1.11	1.67
	1	14.31	28.17	14.52	26.93
	2	36.01	46.87	36.97	47.38
	3	31.34	17.75	31.61	19.79
	>3	17.37	4.01	15.80	4.23
**Verb**					
	0	62.96	83.34	53.09	78.96
	>0	37.04	16.66	46.92	21.05
**Adverb**					
	0	93.86	95.56	91.01	95.38
	>0	6.15	4.45	9.00	4.62
**Adjective**					
	0	54.32	69.71	51.51	66.14
	>0	45.68	30.30	48.50	33.87

## Discussion

### Overview

Increasingly, individuals are actively participating in learning and managing their health by leveraging online resources. The percentage of people using the Internet and the usage of smart devices for health information searching is increasing rapidly. PCs and SDs have very distinct characteristics in terms of readability, user experience, accessibility, etc. These distinct characteristics provide some pros and cons for PCs and SDs: Web browsing and readability are better on PCs while accessibility is better for SDs. Also socioeconomic factors, such as age, gender, income level, education, familiarity with new technologies and devices [4,9], play an important role in the usage of PCs and SDs in general and for online health information seeking. Device characteristics and socioeconomic differences in device usage have an effect on OHISB [4,5,9]. Therefore, in order to improve the health information searching process, it is necessary to understand both aspects, that is, how an OHIS searches for health information and how device choice influences online health information seeking.

In this study, we performed a comparative analysis on the most frequent health search queries launched from PCs and SDs to understand the effects of device type (PCs vs SDs) used for online health information seeking. The analysis dataset consists of search queries between June 2011 and May 2013, which were submitted from Web search engines and directed OHISs to the Mayo Clinic website. The website is visited by millions of unique OHIS every day, and it offers an identical appearance and accessibility for both PCs and SDs using standard Web search engines and Web browsers.

### Principal Results

Following are some of the insights that surfaced from this study. Most of the top search queries from both PCs and SDs are related to symptoms, health conditions, chronic diseases, and diet. Our top search query analysis indicates that the device used has a significant effect on health information searching and the health information searched via different devices is also different (48.49%). While searching for health information, one in every three OHISs searches for “Symptoms”. Other popular health categories that OHISs search for are “Causes” and “Treatments & Drugs”. The analysis suggests that the distribution of search queries for different health categories differs with the device used for health search. Even though most of the diseases can be prevented with some lifestyle and diet changes, very few OHIS search for *preventive* health information. This highlights the fact that we need to promote preventive health care more vigorously.

While searching for health information, OHISs specify words related to women and children more often than that of men and any other age group. The higher percentage of women seeking online health information could be a reason [4,5]. The percentage of OHISs who use words related to “women” and “night” in search queries is higher for PCs than for SDs, while “children” and “morning” are higher for SDs compared to PCs. Health search queries are longer than general search queries, which implies that OHISs describe health information need in more detail. Longer search queries also denote OHIS’s interest in more specific information about the disease; subsequently, OHISs use more words to narrow down to a particular health topic. The average health search query length from SDs is longer than that of PCs, and while typing on SDs is slower and more difficult than typing on PCs, we posit that OHISs might be relying more on Web search engines’ auto-completion functionality, as well as on most devices’ speech recognition facilities, which might be increasing the length of search queries from SDs as compared to that from PCs. These results highlight the differences between usage of PCs and SDs for online health information seeking. The findings can be used by health websites and health application developers to better understand OHISB for PCs and SDs, understand OHIS’s health information needs, and better organize health information content for PCs and SDs users.

For PCs and SDs, 1 in 3 QwoR, and 1 in 10 QwR contained at least one spelling mistake. These mistakes place a burden on the search process and may lead users to incorrect or irrelevant information. The search engine’s auto-completion feature, spelling correction/suggestion, and devices’ speech recognition facilities might be contributing to reducing misspelled words in search queries. Almost all health search queries have at least one noun. In addition to nouns, OHISs use verbs, adverbs, and adjectives while formulating search queries to provide more context for the topic of interest. The percentage of search queries with at least one verb/adverb/adjective is higher for SDs as compared to PCs. This implies that health search queries from SDs are more descriptive as compared to queries from PCs. OHISs formulate search queries by using keywords most frequently, followed by wh-questions and yes/no questions. Considering QwoR, OHIS ask more questions via SD than PC. In wh-questions, OHISs mostly use “What” and “How” in search queries, and both of them generally signify a need for more descriptive information while search queries in the form of yes/no questions indicate interest in factual information.

Since search queries are a fundamental part of health information searching, it is essential that we understand characteristics of health search queries and the role of the device used for searching. This study provides useful insights for online health information retrieval systems. The linguistic structure of a search query has implications in information retrieval using Web search engines [42,43]. Cory Barr et al [42] highlight the importance of recognizing part-of-speech information of the input search query to improve search results and demonstrate that the part-of-speech is a significant feature for information retrieval. Our study provides distribution of part-of-speech in health search queries from PCs and SDs. Expressiveness or descriptiveness of the search queries has a significant impact on quality of the search results using Web search engines [43]. Phan et al [57] specify that with the increase in search query length, the descriptiveness of the query increases. Our study gives basic understanding about health search query descriptiveness based on health query length and part-of-speech analysis. Previous research in information retrieval have identified various important features of search queries such as usage of search query operators [58], misspellings, query length [53-57], query type (keyword-based, wh-questions, yes/no questions), and part-of-speech [42,43]. We presented a comprehensive analysis of these features for health search queries via PCs and SDs.

### Comparison With Related Work

This study contributes a comparative analysis performed on large-scale health search queries to understand the effects of device type (SDs vs PCs) used on OHISB. As discussed in the “Background and Significance” section, previous efforts have used several approaches to understand OHISB including (1) focus groups and user surveys, and (2) analyzing health-related Web search query logs. To the best of our knowledge, there is not much research on understanding the effect of devices on online health search behavior. In our work, we bridge this knowledge gap by analyzing more than 100 million health search queries from PCs and SDs to understand how device choice influences online health information seeking. In addition, we presented analysis for both QwR and QwoR in order to avoid bias from queries with a high number of repetitions. Moreover, we analyzed linguistic structure of health search queries from PCs and SDs, which has implications for Web search engines and information retrieval systems [42,43].

### Limitations

The results of this study are derived from analysis limited to health search queries from Web search engines that led users to Mayo Clinic website. Even though Mayo Clinic web pages often ranked high in Web search engines, not all health information seekers visited the Mayo Clinic website. Also, this analysis is based on the top one million health queries per month (PCs/SDs) rather than the entire health traffic to Mayo Clinic site. In this work, we considered search queries from smartphones and tablets into same categories (ie, smart devices) as the search queries are differentiated based on the operating system of the device used for search, and not the type of specific device per se (eg, Apple iPhone vs iPad vs Android phone). The focus of this study is limited to analysis of a search query log, and we have not analyzed associated socioeconomic factors due to anonymized nature of the data. Previous studies have identified that socioeconomic factors such as age, gender, education, and income have an effect on device usage and OHISB [4,5,9]. Further research in analyzing health search queries based on socioeconomic factors can extend our knowledge about how socioeconomic factors affect health search query formation and the type of health information searched.

### Future Work

In the future, we will extend this work by performing a semantic analysis on the data using biomedical knowledge bases and ontologies. Specifically, we plan to leverage insights from this work and use semantic Web technologies to facilitate health search experience by developing more advanced next-generation knowledge and content delivery systems. Semantic analysis in combination with advanced natural language processing techniques will help us acquire a deeper understanding of OHISB.

### Conclusions

We presented a comprehensive analysis of large-scale health search queries from personal computers (desktops/laptops) and smart devices (smartphones/tablets) in order to understand the effects of device type on online health information search behavior. We noted that online *health* information search behavior differs from *general* online information search. Also, the type of device used for online health information search plays an important role and alters the health information search behavior. A greater understanding of OHIS’s needs, especially how they search and what they search for, may help us understand behavioral changes that will lead to improvement in online health information seeking and a more balanced approach to wellness and prevention. This study extends our knowledge about online health information search behavior and provides useful information for Web search engines, health-centric websites, health care providers, and health care–centric application developers. Finally, we anticipate that this work will help empower OHISs in their quest for health information and facilitate their health information search efforts by enabling the development of more advanced next-generation knowledge and content delivery systems.

## Abbreviations

OHIS: online health information seeker
OHISB: online health information seeking behavior
PC: personal computer (desktop, laptop)
QwoR: queries without considering repetition counts
QwR: queries with considering repetition counts
SD: smart device (smartphone, tablet)
